# First Stage of the Development of an Eco-Friendly Detergent Formulation for Efficient Removal of Carbonized Soil

**DOI:** 10.3390/molecules27217460

**Published:** 2022-11-02

**Authors:** Andreia P. M. Fernandes, Ana M. Ferreira, Marco Sebastião, Ricardo Santos, Catarina M. S. S. Neves, João A. P. Coutinho

**Affiliations:** 1 Mistolin S.A., Zona Industrial de Vagos Lt 58, 3840-385 Vagos, Portugal; 2 CICECO—Aveiro Institute of Materials, Chemistry Department, University of Aveiro, Campus Universitário de Santiago, 3810-193 Aveiro, Portugal

**Keywords:** detergent, degreaser, carbonized soil, cleaning, efficiency, ionic surfactant, nonionic surfactant, solvent, phosphate-free, low pH

## Abstract

Detergent formulations for cleaning a carbonized soil—degreasers—typically comprise surfactants, organic solvents, phosphate-based cleaning agents, and alkaline agents, which results in high pH values (>11) that raise human and environmental risks. It is important to develop eco-friendly and safer degreasers, while maintaining their cleaning efficiency. In this work, simple degreaser formulations, with a pH below 11 and without phosphates, were developed by using a mixture of solvent, surfactant, and water to remove carbonized soil. The efficiency of the new degreaser formulations (with 5 wt% solvent, 5 wt% nonionic or ionic surfactant, and 90 wt% water) was evaluated by an abrasion test in the removal of carbonized soil from ceramic and stainless steel surfaces and compared with a commercial product. The results obtained show that the formulations comprising isopropylene glycol (IPG) with C_11_–C_13_ 9EOs and diethylene glycol butyl ether (BDG) with octyltrimethylammonium octanoate ([N_1118_][C_8_O_2_]) present the best cleaning efficiency for both surfaces. The composition of these formulations was optimized for each surface using a mixture design. The resulting formulations, despite having a simpler composition, a pH lower than 11, and being phosphate-free, presented a cleaning efficiency equal or slightly higher than the commercial control. These results show that it is possible to design degreasers that are much less aggressive to the environment and user, while simultaneously fulfilling the market requirements.

## 1. Introduction

Soiling and cleaning are ubiquitous in the food sector, from the domestic kitchen to large-scale factories, where grease and fatty soils, especially carbonized ones, are difficult to remove [1]. Fat-based soils are usually present as an emulsion, and generally they can be rinsed with hot water above the soil melting point. However, more abrasive detergents are usually required for carbonized residues that are more difficult to remove. These detergents are usually a complex mixture of surfactants, organic solvents, phosphate-based scouring, and alkaline agents (emulsifying/saponifying compounds), which results in an alkaline pH [2]. The use of these compounds and the alkaline medium (pH > 11) in degreaser formulations ensures high efficiency in removing grease, fats, and carbonized food-derived soils [3]. Nevertheless, some of these compounds create risks for human health and environmental pollution [4,5]. Therefore, the development of eco-friendly degreasers (without phosphates and pH < 11) is essential, while maintaining their cleaning efficiency. For that purpose, it is necessary to consider the raw materials used in the degreaser and their function in the formulation [6]. Traditionally, two of the most significant chemicals in degreasers are solvents and surfactants.

The solvents are directly involved in the removal of resins, waxes, adhesives, greases, and paints [7]. They are used in the cleaning industry and are petroleum derivates, including alcohols, amides, amines, esters, glycols, glycol ethers, and hydrocarbons [8,9,10]. Nowadays, there is a demand for biobased solvents, i.e., solvents from renewable sources, such as carbohydrates, carbohydrate polymers, proteins, alkaloids, plant oils, and animal fats to replace petroleum-based ones [7,11]. Companies, such as Solvay, Vertec Biosolvents, and AstroBio, have commercial biosolvents for application in, among others, cosmetics and cleaning products.

Regarding the surfactants, they are present in a wide range of products, such as personal care, lubricants, and detergent formulations, and they are the active ingredient responsible for most of the cleaning power [12,13]. Their main function is lowering a liquid’s surface tension, allowing it to spread over the surface easily, and they adsorb onto the soil, allowing them to remove the soil from the surface into the bulk liquid [14]. It is important to note that after the soil removal, it should be stabilized and suspended (via emulsification and dispersion) in the wash liquor to be rinsed via mechanical agitation [15]. When considering the design of new formulations, surfactant properties, such as molecular weight, critical micelle concentration (CMC), and hydrophilic–lipophilic balance (*HLB*) for the nonionic, must be considered. The surfactants’ molecular weight is an important feature to determine their toxicity and biodegradability [16], mainly due to their alkyl chain length. Generally, surfactants with a low molecular weight have high biodegradability and low toxicity [17].

Aiming at developing an eco-friendly and a safer degreaser for users (pH below 11 and without phosphates) to remove a carbonized soil, a series of simple formulations composed of only one solvent, one surfactant (nonionic or ionic), and water were investigated. For that purpose, first the soil used was characterized by Fourier Transform Infrared—Attenuated Total Reflection (FTIR–ATR), and its surface energy was determined for the two surfaces (ceramic and stainless steel) used in this work. Then, a screening of the individual combinations of 5 wt% solvents and 5 wt% of surfactants was performed using abrasion tests on ceramic and stainless steel surfaces in an attempt to understand which are most effective in removing a particular carbonized soil. In addition to using commercially available nonionic surfactants, a new class of ionic surfactants based on long alkyl trimethylammonium carboxylate was also synthesized. Finally, the best formulations for cleaning carbonized soil were optimized for both surfaces using a mixture design, i.e., to find the optimal percentage of each component (solvent, surfactant, and water) in the formulation for the best cleaning performance. The gathered results show the possibility of having a more benign degreaser for both the user and the environment, with a low pH and phosphate-free, without compromising the market demands regarding the cleaning efficiency of carbonized soil.

## 2. Results and Discussion

### 2.1. Soil Characterization

#### 2.1.1. FTIR–ATR

**Figure 1:**
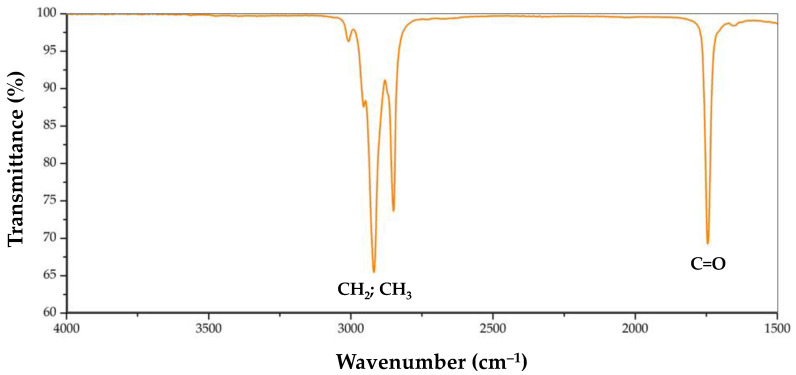
FTIR–ATR spectrum of soil.

In order to characterize the soil used in this work, a FTIR–ATR spectrum of it was obtained as shown in Figure 1. When analyzing the soil spectrum, it is possible to observe a band between 1700 and 1750 cm^−1^, which corresponds to the C=O bond present in the esters and carboxylic acids. The bands that appear between 2800 and 3000 cm^−1^ are the ones of CH_2_ and CH_3_, typical of alkyl chain of esters and carboxylic acids, present in the fats (lard and vegetable oil) used in this soil and paraffin. Moreover, since the soil is carbonized, the typical bands of alcohol and water in the range from 2500 to 3600 cm^−1^, as expected, do not appear.

#### 2.1.2. Surface Energy

**Figure 2:**
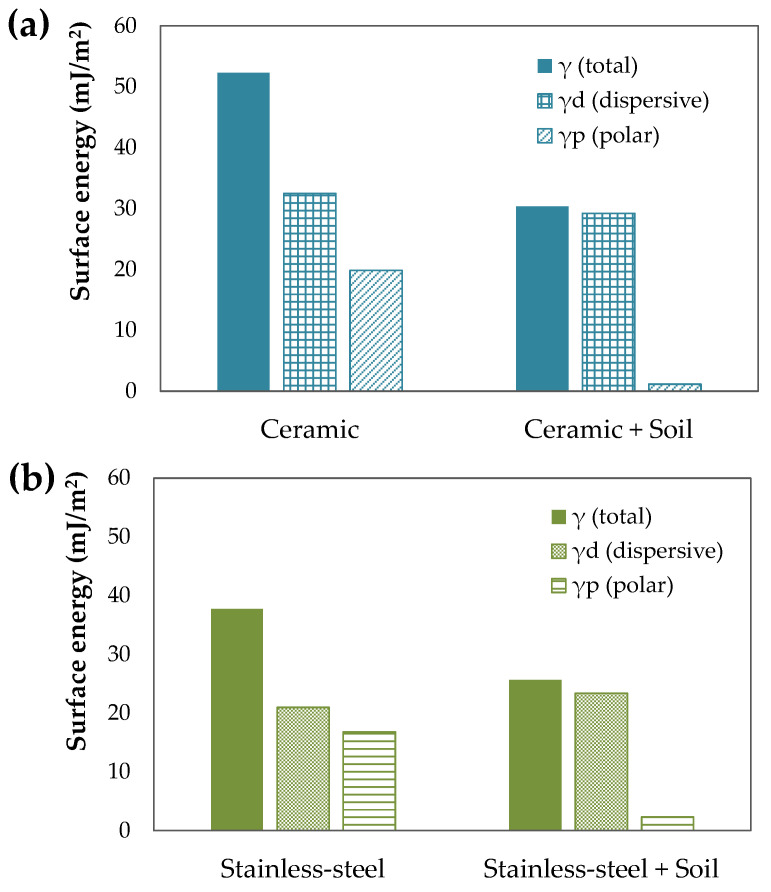
Results of total, dispersive, and polar surface energies on the clean and soiled surfaces: (**a**) ceramic and (**b**) stainless steel.

Figure 2 shows the results obtained for the surface energy for the net and soiled surfaces. The bars represent the total, dispersive, and polar surface energies in which the total surface energy is the sum of the dispersive and polar surface energies.

Comparing the results in Figure 2a, for clean and soiled ceramic surfaces, the soiled one shows the lowest surface energy, making it impermeable, which is in agreement with the composition of the soil (fats). The same phenomenon is observed with stainless steel surfaces (Figure 2b). On both surfaces, the polar component decreases in the presence of soil, showing a surface that is dominated by the dispersive component, as expected due to the high concentration of fats in the soil, whose presence was confirmed by the soil FTIR spectrum in Figure 1.

### 2.2. Evaluating the Efficiency of the New Formulations

**Figure 3:**
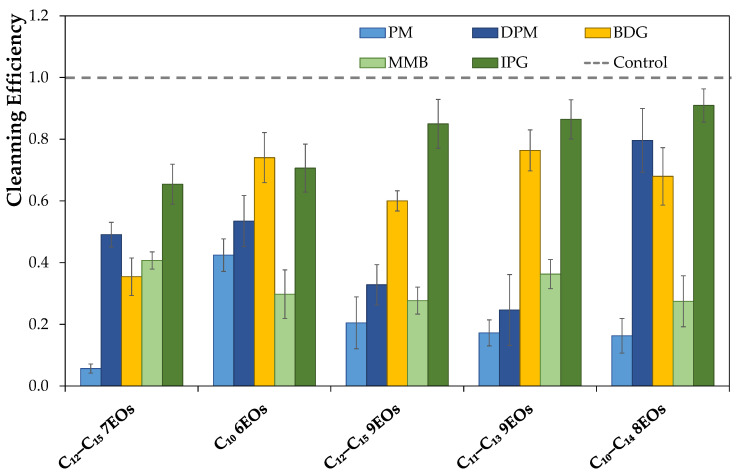
Cleaning efficiency of formulations with nonionic surfactants in ceramic tiles obtained by abrasion scrub test: bars represent the cleaning efficiency of the formulation with different solvents, and error bars are the standard deviation (between 1% and 11%) of at least five independent measurements.

**Figure 4:**
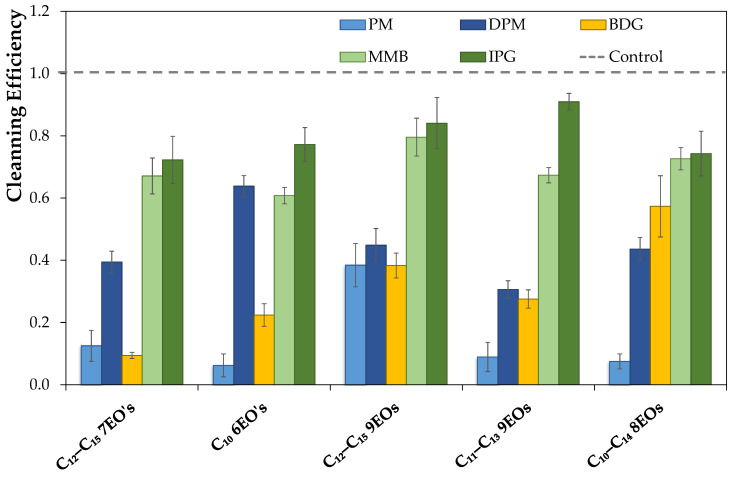
Cleaning efficiency of formulations with nonionic surfactants on stainless steel surface obtained by abrasion scrub test: bars represent the cleaning efficiency of the formulation with different solvents, and error bars are the standard deviation (between 1% and 10%) of at least five independent measurements.

**Figure 5:**
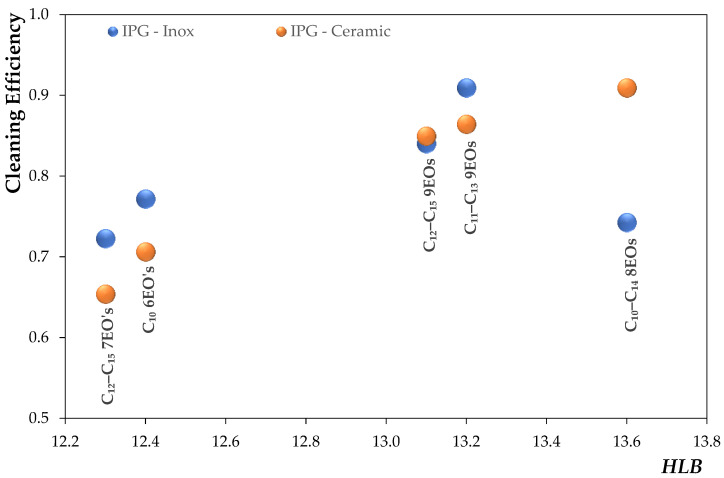
Cleaning efficiency vs. *HLB* of formulations with IPG as solvent and nonionic surfactants on both surfaces: ceramic and stainless steel.

**Figure 6:**
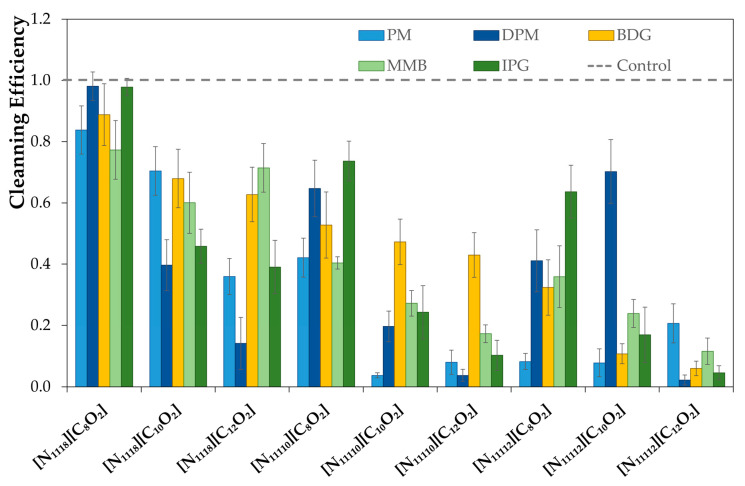
Cleaning efficiency of formulations with ionic surfactants in ceramic tiles obtained by abrasion scrub test: bars represent the cleaning efficiency of the formulation with different solvents, and error bars are the standard deviation (between 1% and 11%) of at least five independent measurements.

**Figure 7:**
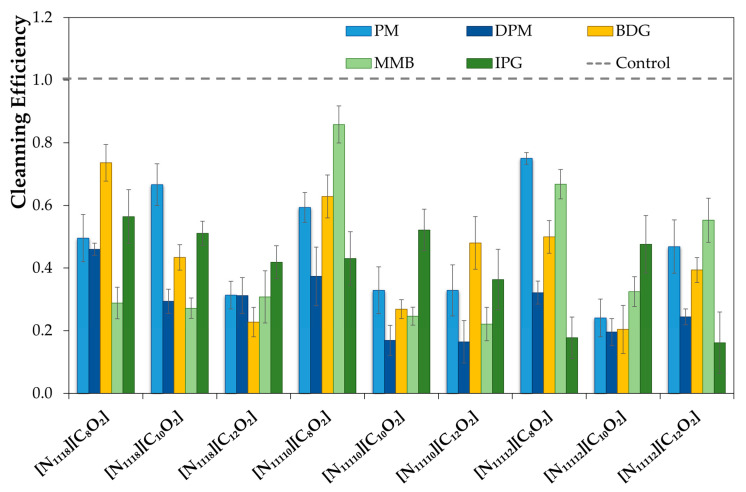
Cleaning efficiency of formulations with ionic surfactants on stainless steel surface obtained by abrasion scrub test: bars represent the cleaning efficiency of the formulation with different solvents, and error bars are the standard deviation of at least five independent measurements.

The new formulations comprise the different combinations of all solvents and surfactants selected for this work, in a concentration of 5 wt% each and 90 wt% water. Since the pH of the formulation is an important parameter, it was determined for all formulations, and the values obtained range between 3.2 and 10.4 (*cf* Supplementary Materials, Table S1). These results show that the solution pH is mainly influenced by the surfactant used, where the solutions with nonionic surfactants are acidic (pH values between 3 and 6) and those with ionic surfactants are alkaline (pH values from 7.5 to 10.5). The cleaning efficiency of the developed formulations on both surfaces are presented in Figure 3, Figure 4, Figure 5, Figure 6 and Figure 7.

#### 2.2.1. Formulations Based on Nonionic Surfactants

Regarding the formulations with nonionic surfactants (Figure 3), those that stand out are based on the solvent IPG in combination with C_12_–C_15_ 9EOs, C_11_–C_13_ 9EOs, and C_10_–C_14_ 8EOs with cleaning efficiencies between 0.85 and 0.91. These surfactants have *HLB* values ranging from 13.1 for C_12_–C_15_ 9EOs to 13.6 for C_10_–C_14_ 8EOs (the *HLB* values are given by the supplier). The high *HLB* of these nonionic surfactants explains their high solubility in water, and, in combination with the solvent, they allow better soil removal on ceramics. On the other hand, formulations with propylene glycol methyl ether (PM) solvent show poor results, with cleaning efficiencies below 0.42.

Figure 4 presents the results of formulations with nonionic surfactants for soil removal from stainless steel. The gathered results show that these formulations are more effective with solvents, such as 3-methoxy-3-methyl-1-butanol (MMB) and IPG. The most efficient formulation tested comprises IPG and C_11_–C_13_ 9EOs, being only 9% less efficient than the control. Once again, the combination of nonionic surfactants and PM was poorly effective in the soil removal, with a cleaning efficiency below 0.40.

The results presented in Figure 5 suggest that for the formulations with IPG and nonionic surfactants the best cleaning performance is achieved for the formulations with surfactants with higher *HLB* values. The only exception is for the formulation with the IPG and C_10_–C_14_ 8EO surfactant, which significantly decreases its cleaning efficiency.

#### 2.2.2. Formulations Based on Ionic Surfactants

In Figure 6, the ionic surfactants are ordered first by the cation alkyl chain length (hydrophilic head of the surfactant) and then by the anion hydrocarbon chain (hydrophobic tail of the surfactant). When comparing ionic surfactants, it is clear that formulations with a surfactant with a shorter alkyl chain length both in the cation and anion (trimethyloctylammonium octanoate, [N_1118_][C_8_O_2_]) are more efficient in removing the soil from ceramic tiles than the most hydrophobic ones, with longer alkyl chains (dodecyltrimethylammonium dodecanoate, [N_11112_][C_12_O_2_]). From the solvents used, dipropylene glycol methyl ether (DPM) and IPG, in combination with [N_1118_][C_8_O_2_], reached a cleaning efficiency similar to the control (0.98). On the other hand, these solvents presented the worst results in combination with the surfactant [N_11112_][C_12_O_2_] (0.022 and 0.045 for DPM and IPG, respectively).

The same formulations were used to test their cleaning efficiency on stainless steel, and the results obtained are depicted in Figure 7. The soil removal results on stainless steel surfaces show different behaviors and trends compared to the ceramic tile results. This fact is due to the soil’s strong adhesion to the stainless steel surface after cooking, being therefore more challenging to remove. Even with less removal capacity, there was one formulation that stood out from the others with an ionic surfactant, which contains the solvent MMB and the surfactant decyltrimethylammonium octanoate ([N_11110_][C_8_O_2_]), with a cleaning efficiency of 0.86. The remaining formulations with ionic surfactants have cleaning efficiency values that range from 0.29 to 0.78.

Although the results presented here do not reach the efficiency of the commercial degreaser used as the control, the formulations used are much simpler than those in the commercial products and yet unoptimized. Moreover, all the formulations have a lower pH than the commercial products currently available, reducing the risks for the user and minimizing the hazards in handling and transportation.

### 2.3. Optimization of the Formulation Composition

**Figure 8:**
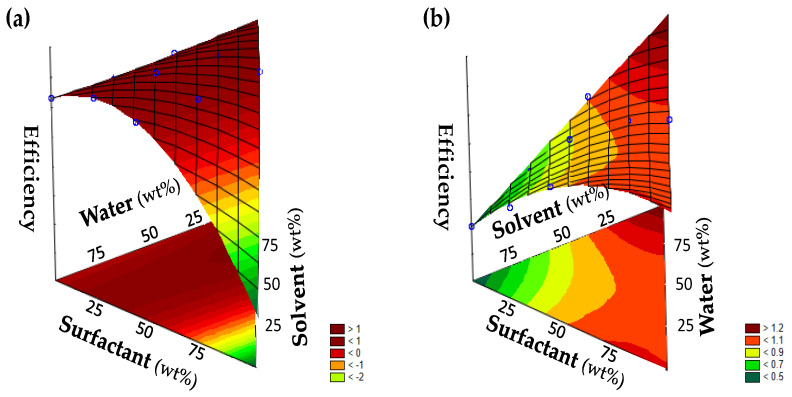
Response surface of cleaning efficiency for (**a**) ceramic and (**b**) stainless steel as a function of the composition of the ternary mixture in weight percentage (IPG (solvent), C_11_–C_13_ 9EOs (surfactant), and water).

**Figure 9:**
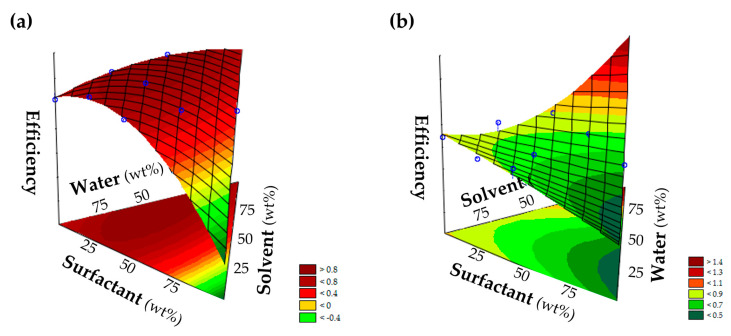
Response surface of cleaning efficiency for (**a**) ceramic and (**b**) stainless steel as a function of the composition of the ternary mixture in weight percentage (BDG (solvent), [N_1118_][C_8_O_2_] (surfactant), and water.

Considering the aforementioned results, two formulations, one with an ionic surfactant and the other with a nonionic one, stood out from the others leading to a high soil removal efficiency from both surfaces when compared to the remaining formulations. The results in Figure 7 and Figure 8 show that the formulations [N_11110_][C_8_O_2_] + MMB and [N_1118_][C_8_O_2_] + (DPM or IPG) have better results for the stainless steel and ceramic surfaces, respectively. However, BDG + [N_1118_][C_8_O_2_] was identified as the best-performing formulation for all surface types. Regarding the formulations with nonionic surfactants, the best ones were composed of IPG + (C_12_–C_15_ 9EOs or C_11_–C_13_ 9EOs), and the IPG + C_11_–C_13_ 9EOs was selected due to its best performance on both surfaces. Therefore, two ternary mixture designs were carried out, one using IPG + C_11_–C_13_ 9EOs + water and another using BDG + [N_1118_][C_8_O_2_] + water, in order to find the best formulation composition that allows to achieve a high cleaning efficiency. The mixture designs of the formulations were performed on both surfaces in the study (Tables S3 to S5), and the results obtained can be seen in Figure 8 and Figure 9. All analyses were carried out with a confidence level of 95% using the statistical model analysis variance (ANOVA) shown in the Supplementary Materials (Figures S3–S5). The experimental and predicted results are very similar (all analyses present an R^2^ > 0.80), showing the statistical model used to be adequate.

#### 2.3.1. Formulation Based on a Nonionic Surfactant

Figure 8 presents the ternary diagrams of IPG, C_11_–C_13_ 9EOs, and water mixtures for ceramic and stainless steel surfaces and the corresponding cleaning efficiency values. All experimental data are detailed in Table S4. Models were fit using a quadratic model, with a 95% confidence level. The ANOVA analysis was also evaluated (Tables S6 and S7), and it was found that the F-values of the models were highly significant; the P-values were less than 0.05, and the values of SSresidual were relatively small compared with the SSregression. In addition, R^2^ values of 0.97 and 0.8 were obtained for the ceramic and stainless steel surfaces, respectively. Therefore, the statistical analysis shows a good agreement between the predicted values by the model and the experimental ones, as shown in Figure 8.

According to Figure 8 and Figures S2 and S3 in the Supplementary Materials, the concentrations of the active ingredients, and in particular of the solvent, were the most significant parameters for both surfaces. The optimized formulation composition that maximized the cleaning efficiency for the ceramic surface was 10 wt% of IPG, 5 wt% of C_11_–C_13_ 9EOs, and 85 wt% of water. A similar formulation composition was found for the stainless steel surface: 13 wt% of IPG, 3 wt% of C_11_–C_13_ 9EOs, and 84 wt% of water. At these compositions, the model predicted values of 1.03 and 0.95 (Table S8) and the experimentally efficiency was found to be 1.00 and 0.91 for ceramic and stainless steel, respectively, which demonstrates the model’s predictive ability. The nonionic surfactant formulation seems more effective in cleaning ceramic surfaces than stainless steel. Furthermore, the optimized formulations do not present stability problems since the solutions were colorless (transparent) and homogeneous at room temperature even after 4 months. It should be also highlighted that the pH value of the optimized formulations was about 4, demonstrating that it is possible to develop a degreaser with high cleaning efficiency without a high pH (>11).

#### 2.3.2. Formulations Based on an Ionic Surfactant

The influence of the three components (BDG, [N_1118_][C_8_O_2_], and water) on the cleaning efficiency on both surfaces is presented in Figure 9, while more details can be found in Table S5 and Figures S4 and S5 in the Supplementary Materials. According to Pareto Chart (Figure S5) and data depicted in Figure 9, for the ceramic surface, interaction variables (especially water–surfactant and solvent–surfactant) followed by surfactant and water are the most significative variables for the cleaning efficiency, while for the stainless steel surface, the most significates ones were water and solvent. Maximum response values were observed at high concentrations of water; these were also observed for the nonionic formulation, but in this case, for values as low as 75 wt%. The optimal conditions obtained for ceramic surfaces were 13% solvent, 7% surfactant, and 80 wt% of water, while for stainless steel surfaces, they were were 13% solvent, 8% surfactant, and 79 wt% of water. The experimental efficiency at these optimum compositions was 0.99 for ceramic surfaces and 1.04 for stainless steel, and as previously observed for the formulation based on a nonionic surfactant, there was also good agreement here between the experimental and predicted data for formulation based on the ionic surfactant (Table S7) revealing a good predictive ability of the models. Thus, the optimized formulations presented a cleaning efficiency to remove carbonized soil equal to or slightly higher than the control used, with a pH value around 8. Moreover, the results obtained for the optimization of the formulation based on an ionic surfactant are very promising, since both formulations present a similar optimal composition for soil removal, meaning these formulations have the ability to clean different types of surfaces. In addition, the formulation based on a nonionic surfactant seems to be more effective in cleaning stainless steel surfaces, since the cleaning efficiencies for this surface were a little higher (1.04) than for the ceramic surface (0.99). Again, the optimized formulations were colorless (transparent) and homogeneous at room temperature even after 4 months, which shows their stability.

## 3. Materials and Methods

### 3.1. Materials

In this work, the solvents used to test new formulations, namely propylene glycol methyl ether (PM), dipropylene glycol methyl ether (DPM, 1-((1-methoxypropan-2-yl)oxy)propan-2-ol), diethylene glycol butyl ether (BDG), and 3-methoxy-3-methyl-1-butanol (MMB) were supplied by Kuraray CO (Tokyo, Japan), while isopropylene glycerol (IPG) was kindly provided by Solvay (Paris, France). The nonionic surfactants used were C_10_ 6EOs, C_11_–C_13_ 9EOs, and C_12_–C_15_ 7EOs that were kindly supplied by Sasol (Milan, Italy) and KAO (Barcelona, Spain), C_12_–C_15_ 9EOs that was kindly provided by Evonik (Essen, Germany), and C_10_–C_14_ 8EOs, that was kindly provided by Huntsman (Freeport, TX, USA). In order to synthesize the new ionic surfactants, the starting materials octyltrimethylammonium bromide (98.0%, from TCI, Tokyo, Japan), decyltrimethylammonium bromide (98.0%, from TCI, Tokyo, Japan), decyltrimethylammonium chloride (98.0%, from TCI, Tokyo, Japan), dodecyltrimethylammonium bromide (99.0%, from Alfa Aesar, Kandel, Germany), sodium octanoate (98.0%, from Acros Organics, India), sodium decanoate (98.0%, from Sigma, USA), and sodium dodecanoate (98.0%, from Acros Organics, China) were used.

The soil used in the abrasion tests comprised lard, vegetable oil, and sugar powder (all acquired in a local supermarket, Portugal); paraffin wax (purum, from Aldrich, Germany), carbon black (from Kremer Pigmente, Aichstetten, Germany), and albumin powder from egg (from Acros Organics, Poland). All new formulations were compared with the commercial detergent KH7 degreaser from the company KH Lloreda (details in Suplementary Materials), purchased in a local supermarket, hereafter denominated as control.

### 3.2. FTIR-ATR

The soil FTIR (Fourier-transform infrared) spectra were taken with a PIKE MIRacle MB3000 FTIR System spectrometer equipped with an ATR (attenuated total reflection) cell with a ZnSe crystal. The resolution was 4 cm^−1^ after 40 scans, and the spectra were collected from 4000 to 600 cm^−1^.

### 3.3. Surface Energy and Contact Angle Measurements

For the soil characterization, contact angle and surface energy were measured. Contact angle measurements of the liquids water (MilliQ grade), formamide (Sigma, 99% purity GC), and diiodomethane (Aldrich, 99% purity GC) were carried out using the sessile drop method with the Contact Angle System OCA 20 (DataPhysics Instruments GmbH, Germany) at room temperature. Drop volumes of (1 ± 0.01) µL were obtained using a Hamilton DS 500/GT syringe connected to a Teflon-coated needle placed inside an aluminum chamber. The reported contact angles of each standard were an average of at least ten independent measurements.

The surface energy can be used to describe the wettability/permeability. The total surface energy ($\gamma_{s}$) of the soil, ceramic, and stainless steel was obtained by the Owens–Wendt–Rabel–Kaelble (OWRK) model [18], and the corresponding polar ($\gamma_{s}^{p}$) and dispersive ($\gamma_{s}^{d}$) components by measuring contact angles (*θ)*, liquid-probe surface tension (Table 1) data and Equations (1) and (2):

(1) $$\gamma_{s}=\gamma_{s}^{d}+\gamma_{s}^{p}$$

(2) $$\frac{\left(1+\cos\theta \right)}{2}\times \frac{\gamma_{l}}{\sqrt{\gamma_{l}^{d}}}=\sqrt{\gamma_{s}^{p}}\times \sqrt{\frac{\gamma_{l}^{p}}{\gamma_{l}^{d}}}+\sqrt{\gamma_{s}^{d}}\;$$

where $\gamma_{L}$ is the total surface tension of the liquid (mN/m); *θ* is the contact angle (radians), and $\gamma_{l}^{d}$ and $\gamma_{s}^{d}$ are the dispersive components, and $\gamma_{s}^{p}$ and $\gamma_{l}^{p}$ are the polar components of solid and liquid surface energies, respectively. Plotting the right-hand side of Equation (2) as a function of $\sqrt{\frac{\gamma_{l}^{p}}{\gamma_{l}^{d}}}$ enables the calculation of $\gamma_{s}^{p}$ and $\gamma_{s}^{d}$ from the parameters of the linear regression (Equation (2)).

### 3.4. Synthesis of Ionic Surfactants

**Scheme 1:**
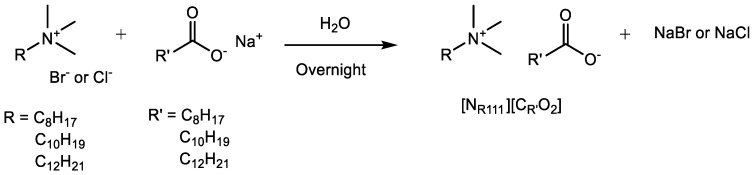
Synthetic route of ionic surfactants. Different combinations between R and R’ were performed resulting in the ionic surfactants: [N_1118_][C_8_O_2_], [N_1118_][C_10_O_2_], [N_1118_][C_12_O_2_], [N_11110_][C_8_O_2_], [N_11110_][C_10_O_2_], [N_11110_][C_12_O_2_], [N_11112_][C_8_O_2_], [N_11112_][C_10_O_2_], and [N_11112_][C_12_O_2_].

The synthesis of new ionic surfactants consists of a salt metathesis reaction described in Scheme 1. Chloride or bromide salt and the corresponding sodium salt were used to synthesize these ionic compounds in the stoichiometry of 1:1. The reaction occured in water, under stirring for at least 12 hours. Then, the water was removed in a moderate vacuum (0.1 Pa) for at least 48 hours, and the surfactant structures were checked by ^1^H and ^13^C Nuclear Magnetic Ressonance (NMR) spectroscopy (*cf* Figure S6 from Supplementary Materials). This is were the different combinations of the sodium and chloride or bromide salts were performed, resulting in the surfactants octyltrimethilammonium octanoate ([N_1118_][C_8_O_2_]), octyltrimethilammonium decanoate ([N_1118_][C_10_O_2_]), octyltrimethilammonium dodecanoate ([N_1118_][C_12_O_2_]), decyltrimethilammonium octanoate ([N_11110_][C_8_O_2_]), decyltrimethilammonium decanoate ([N_11110_][C_10_O_2_]), decyltrimethilammonium dodecanoate ([N_11110_][C_12_O_2_]), dodeciltrimethilammonium octanoate ([N_11112_][C_8_O_2_]), dodeciltrimethilammonium decanoate ([N_11112_][C_10_O_2_]), and dodeciltrimethilammonium dodecanoate ([N_11112_][C_12_O_2_]). Moreover, the surfactant was used without a purification step to remove the sodium chloride or bromide salt produced as a secondary product from the synthesis.

### 3.5. New Formulations

**Figure 10:**
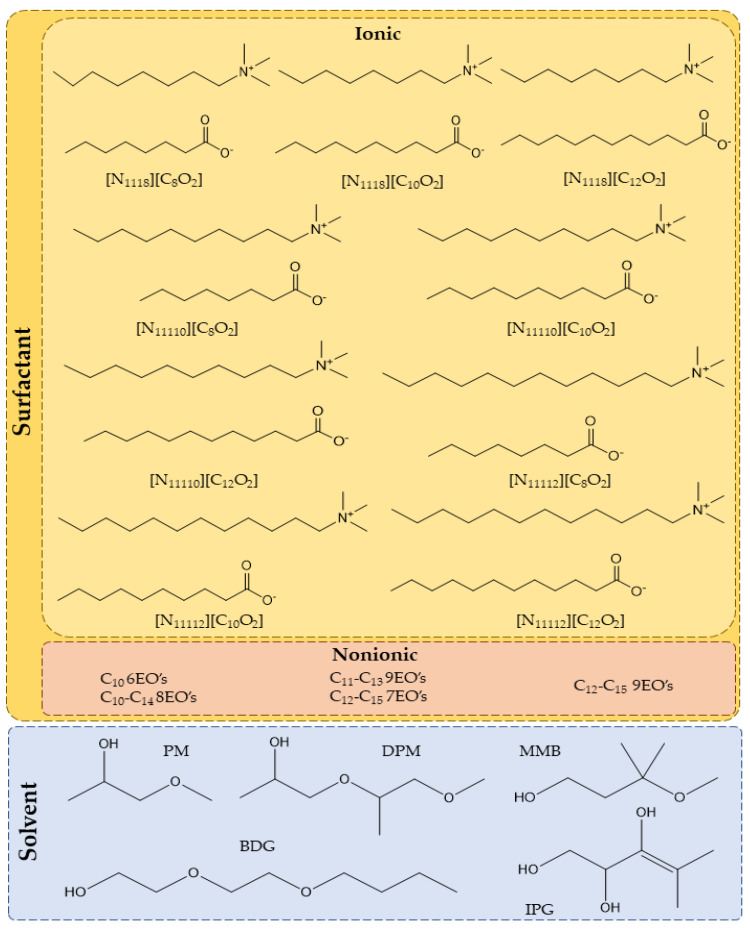
Chemical structures of solvents and surfactants used in the development of degreaser formulations.

After the synthesis of surfactants, different combinations between the solvents and surfactants were tested for new formulations. The chemical structures of solvents and surfactants used are presented in Figure 10. The composition of new formulations comprised 5 wt% solvent, 5 wt% surfactant, and 90 wt% distilled water. The pH of each formulation was measured by a SevenExcellence pH meter from Mettler Toledo^™^ (Columbus, OH, USA).

### 3.6. Wet Abrasion Scrub Test

To test the new formulations, an abrasion test was conducted using a Sheen^®^ 903 Wet Abrasion Scrub Tester against a soil previously applied to the ceramic and stainless steel surface. The soil used, adapted from the literature [20], comprised lard, paraffin, carbon black, vegetable oil, sugar powder, and albumin powder at different proportions. For the soil preparation, lard, paraffin, and vegetable oil were first mixed under stirring at 90 °C, then, the carbon black and the albumin were added. Next, the temperature was increased up to 110 °C, and the sugar powder was added. Subsequently, the heat was turned off while stirring it until the mixture cooled down to room temperature, and was afterward stored in a humidity-controlled chamber for three days before use. The soil was then applied to the different surfaces using a stencil, which guaranteed an even application. Then, the surfaces were cooked at 185 °C for 15 minutes, and after that, stored in a humidity-controlled chamber overnight.

To test the formulations, the surfaces and sponges (with 15 g of formulation) were placed in the Sheen^®^ 903 Wet Abrasion Scrub Tester. A few swipes (back and forward movement is one swipe) were determined as the minimum number required to remove 70% of the soil from the surface using a control formulation. Then, the tests with new formulations were performed with the same number of swipes.

In order to analyze the cleaning potential of each formulation in the ceramic tiles, a black and white photograph was taken before and after the cleaning procedure, using GenoSmart2 Image Capture equipment from VWR. To quantify the soil removal in each tile, a Matlab routine was used to analyze the photograph on a scale of gray. From it, a histogram considering the number of pixels for each gray tone was plotted, and the results were analyzed taking into consideration the mean (*µ*) of the histogram, which is given by the following equation:

(3) $$\mu =\frac{1}{N}\sum_{i=0}^{N=1}Z_{i}=\sum_{k=0}^{K=1}kf\left(k\right)\;$$

where *f*(*k*) is the histogram; *k* is the bin index (gray level), and *K* is the number of gray levels. The difference in the means of the distribution of the histogram representing the pictures before and after cleaning (in absolute values) gave the cleaning performance of each formulation. Another method was used for the stainless steel surfaces since the contrast of soil and surfaces was not adequate to apply the process previously described. Therefore, a weighting method was used to quantify the cleaning efficiency by weighting the surface before and after the cleaning procedure, in which the cleaning efficiency was determined by the soil mass removed. Each formulation was tested with at least five surfaces for both methods, and the cleaning value obtained was the average of those values. Finally, all the results were normalized with the control value and have the standard deviations presented as error bars in the data analysis.

### 3.7. Mixture Design

Mixture design is a tool to study synergistic and antagonistic effects among components and determine the optimal formulation composition, through experimental planning [21]. This study aimed to obtain the optimal concentration of the formulation components (independent variables) to reach the maximum cleaning efficiency (dependent variable). In this work, the formulations were a mixture of three components (surfactant, solvent, and water) with concentrations ranging from 3 wt% to 10 wt% of surfactant, 3 wt% to 13 wt% of solvent, and from 77 wt% to 94 wt% of water. For each formulation, nine experiments were developed, and the concentrations applied are provided in the Supplementary Materials (Table S3). To design this mixture, the statistic and data analysis software JMP was used. The obtained results were statistically analyzed with a confidence level of 95% and subjected to analysis of variance (ANOVA) and regression analysis using the Statistica 7.0 software (StatSoft, Tulsa, OK, USA). The response surface of the concentrations was generated from adjusted models, and the optimal conditions could be determined through their analysis.

## 4. Conclusions

In this work, we attempted to develop new detergent formulations that could clean a carbonized soil from ceramics and stainless steel surfaces with a pH much lower than the current pH that most of the available commercial products use, which constitutes a risk for the user and the environment. A screening of a large number of combinations of solvents and ionic and nonionic surfactants was carried, and the formulations IPG + C_11_–C_13_ 9EOs and BDG + [N_1118_][C_8_O_2_] stood out. The formulation composition was then optimized by experimental design, and the results show a cleaning efficiency equal or slightly higher than the control. For the formulation composed of IPG and C_11_–C_13_ 9EOs, the optimal composition was 10–15 wt% solvent and 3–5 wt% surfactant, presenting a pH of around 4, while the formulation with BDG and [N_1118_][C_8_O_2_] showed an optimal composition of 13 wt% solvent and 7–8 wt% surfactant, with a pH of approximately 8. Moreover, nonionic surfactants showed better efficiencies in removing the carbonized soil from ceramic surface (cleaning efficiency of 1.00), and ionic surfactants showed better efficiencies on stainless steel surfaces (cleaning efficiency of 1.04). The use of IPG as a solvent is a more sustainable approach to degreaser formulations since it is a biobased solvent derived from renewable sources. This work shows that it is possible to develop simple formulations with environmentally friendly and sustainable compounds, with lower pH values. The results presented here produce new perspectives on using more sustainable compounds in the development of degreaser formulations while removing the necessity of using phosphates and high alkaline conditions.

## Figures and Tables

**Table 1:** Surface tension ($\gamma_{l}$ ) and corresponding polar ($\gamma_{l}^{p}$ ) and dispersive ($\gamma_{l}^{d}$ ) components of the standard liquids used for surface energy calculations [18,19].

Liquid	$\gamma_{l}$ (mJ/m^2^)	$\gamma_{l}^{p}$ (mJ/m^2^)	$\gamma_{l}^{d}$ (mJ/m^2^)
Water	72.8	51.0	21.8
Formamide	58.0	20.4	37.6
Diiodomethane	50.8	2.3	48.5

## Data Availability

Not applicable.

## Supplementary Materials

The following supporting information can be downloaded at: https://www.mdpi.com/article/10.3390/molecules27217460/s1, Table S1: pH of the different formulations used in the screening; Table S2: *HLB* values of nonionic surfactants, given by the suppliers; Table S3: Mixture design for optimization of the solvent composition; Table S4: pH and efficiency obtained for the formulations used in the mixture design of C_11_–C_13_ 9EOs, IPG, and water; Table S5: pH and efficiency obtained for the formulations used in the mixture design of [N_1118_][C_8_O_2_], BDG, and water; Table S6: ANOVA for the mixture design using formulations composed of C_11_–C_13_ 9EOs, IPG and water applied to the ceramic surface; Table S7: ANOVA for the mixture design using formulations composed of C_11_–C_13_ 9EOs, IPG and water applied to the stainless-steel surface; Table S8: Efficiency and pH obtained for the optimal composition of the formulation composed of C_11_–C_13_ 9EOs, IPG, and water; Table S9: ANOVA for the mixture design using formulations composed of [N_1118_][C_8_O_2_], BDG and water applied to the ceramic surface; Table S10. ANOVA for the mixture design using formulations composed of [N_1118_][C_8_O_2_], BDG and water applied to the stainless-steel surface; Table S11: Efficiency and pH obtained for the optimal composition of the formulation composed of [N_1118_][C_8_O_2_], BDG, and water; Figure S1: Contact angles of net and soiled surfaces: (a) ceramic and (b) stainless steel; Figure S2: Predicted vs. observed values of IPG + C_11_–C_13_ 9EOs for soil removal from (A) ceramic and (B) stainless steel; Figure S3: Pareto charts for the standardized main effects in the IPG + C_11_–C_13_ 9EO mixture design for (A) ceramic and (B) stainless steel. The vertical line indicates the statistical significance of the effects (95% of confidence); Figure S4: Predicted vs. observed values of BDG + [N_1118_][C_8_O_2_] for soil’s removal from (A) ceramic and (B) stainless steel; Figure S5: Pareto charts for the standardized main effects in the BDG + [N_1118_][C_8_O_2_] mixture design for (A) ceramic and (B) stainless steel. The vertical line indicates the statistical significance of the effects (95% of confidence); Figure S6: ^1^H and ^13^C NMR spectra of the ionic surfactants synthesized.

## Sample Availability

Samples of the compounds are not available from the authors.
