# Supplementary material for: First Stage of the Development of an Eco-Friendly Detergent Formulation for Efficient Removal of Carbonized Soil
Source: Molecules. 2022 Nov 2;27(21):7460. doi: 10.3390/molecules27217460 (PMC9656870; doi:10.3390/molecules27217460)
Supplement: Supplementary file 1 [file molecules-27-07460-s001.zip › molecules-1935237-Supplementary.pdf]

Supplementary Materials

First Stage of the Development of an Eco-Friendly Detergent Formulation for Efficient Removal of Carbonized Soil

Andreia P. M. Fernandes[1], Ana M. Ferreira[2], Marco Sebastião[1], Ricardo Santos[1], Catarina M. S. S. Neves[2*] and João A.P. Coutinho[2]

[1]*Mistolin S.A., Zona Industrial de Vagos Lt 58, 3840-385 Vagos, Portugal.*

[2]*CICECO – Aveiro Institute of Materials, Department of Chemistry, University of Aveiro, 3810-193, Aveiro, Portugal.*

*Corresponding author: catarinasn@ua.pt

Contents

Number of pages: 14

Number of figures: 6

Number of tables: 11

In this work, the commercial degreaser KH7, from KH Lloreda company, was used. This degreaser is composed of water, fatty ethoxylated alcohol, methoxypropanol, monoethanolamine and polycarboxylate with a pH of 11.25.

Tables

Table S1. pH of the different formulations used in the screening.

	pH				
**Solvent →** **Surfactant ↓**	PM	DPM	BDG	MMB	IPG
$[N_{1118}][C_8O_2]$	10.36	7.91	8.53	8.54	10.26
$[N_{1118}][C_{10}O_2]$	8.87	8.20	8.28	8.28	8.75
$[N_{1118}][C_{12}O_2]$	9.02	8.48	9.06	8.84	8.97
$[N_{11110}][C_8O_2]$	10.19	8.32	9.82	7.86	10.38
$[N_{11110}][C_{10}O_2]$	9.21	8.45	8.83	8.83	9.06
$[N_{11110}][C_{12}O_2]$	9.30	8.56	8.33	8.10	9.34
$[N_{11112}][C_{10}O_2]$	9.35	8.39	8.75	9.15	9.25
$[N_{11112}][C_{12}O_2]$	9.65	8.62	9.00	9.30	9.46
C_{12}-C_{15} 7EO’s	5.08	4.15	4.99	4.05	5.11
C_{10} 6EO's	4.68	4.04	4.67	3.18	4.54
C_{12}-C_{15} 9EO's	5.80	4.30	5.94	4.19	5.43
C_{11}-C_{13} 9EO’s	5.07	3.95	4.99	3.90	4.71
C_{10}-C_{14} 8EO's	5.10	4.05	4.86	3.98	5.04

Table S2. *HLB* values of nonionic surfactants, given by the suppliers.

Nonionic surfactant	*HLB*
C_{10} 6EO’s	12.4
C_{11}-C_{13} 9EO’s	13.2
C_{12}-C_{15} 7EO’s	12.3
C_{12}-C_{15} 9EO's	13.1
C_{10}-C_{14} 8EO's	13.6

Table S3. Mixture design for optimization of the solvent composition.

Run	Coded variables		
	Surfactant (wt%)	Solvent (wt%)	Water (wt%)
1	10.00	13.00	77.00
2	10.00	3.00	87.00
3	3.00	13.00	84.00
4	3.00	3.00	94.00
5	3.00	8.00	89.00
6	10.00	8.00	82.00
7	6.50	3.00	90.50
8	6.50	13.00	80.50
9	6.50	8.00	85.50

Table S4. pH and efficiency obtained for the formulations used in the mixture design of C_{11}-C_{13} 9EO's, IPG and water.

Model for ceramic: $R^2 = 0.97$ and $R^2_{adj.} = 0.91$

Model for stainless-steel: $R^2 = 0.80$ and $R^2_{adj} = 0.47$

Run	pH	Experimental Efficiency		Predicted Efficiency	
		Ceramic	Stainless-steel	Ceramic	Stainless-steel
1	4.24	0.45	0.65	0.42	0.61
2	4.09	0.76	0.87	0.79	0.80
3	4.24	1.02	0.87	0.99	0.93
4	4.18	0.60	0.90	0.62	0.92
5	4.19	0.90	0.90	0.90	0.83
6	4.15	0.70	0.51	0.70	0.61
7	4.30	0.93	0.82	0.87	0.86
8	4.20	0.82	0.80	0.87	0.78
9	4.14	0.96	0.74	0.97	0.72

Table S5. pH and efficiency obtained for the formulations used in the mixture design of $[N_{1118}][C_8O_2]$, BDG and water.

Model for ceramic: $R^2 = 0.94$ and $R^2_{adj.} = 0.84$

Model for stainless-steel: $R^2 = 0.92$ and $R^2_{adj} = 0.80$

Run	pH	Experimental Efficiency		Predicted Efficiency	
		Ceramic	Stainless-steel	Ceramic	Stainless-steel
1	8.23	0.87	1.10	0.83	1.06
2	8.74	0.63	0.95	0.65	0.93
3	7.58	0.47	1.03	0.41	0.99
4	7.92	0.32	0.41	0.31	0.40
5	7.70	0.25	0.65	0.33	0.70
6	8.41	0.68	0.93	0.70	0.99
7	7.83	0.87	0.68	0.87	0.71
8	8.05	0.90	0.98	1.00	1.06
9	8.15	1.00	1.00	0.90	0.89

Table S6. ANOVA for the mixture design using formulations composed of C_{11}-C_{13} 9EOs, IPG and water applied to the ceramic surface.

	SS	df	MS	F	p
**Model (Regression)**	0.265929	5	0.053186	17.30503	0.020203
**Total Error (Residual)**	0.009220	3	0.003073		
**Total Adjusted**	0.275149	8	0.034394		

Table S7. ANOVA for the mixture design using formulations composed of C_{11}-C_{13} 9EOs, IPG and water applied to the stainless-steel surface.

	SS	df	MS	F	p
**Model (Regression)**	0.110659	5	0.022132	3.399777	0.051053
**Total Error (Residual)**	0.027667	3	0.009222		
**Total Adjusted**	0.138327	8	0.017291		

Table S8. Efficiency and pH obtained for the optimal composition of the formulation composed of C_{11}-C_{13} 9EO's, IPG and water.

Surface	Optimal composition			Efficiency		Relative deviation (%)	pH
	IPG	C_{11}-C_{13} 9EO's	H_2O	Experimental	Predicted		
Ceramic	10.0	5.0	85.0	1.03	1.00	3.00	4.30
Stainless-steel	13.0	3.0	84.0	0.95	0.91	4.40	4.24

Table S9. ANOVA for the mixture design using formulations composed of $[N_{1118}][C_8O_2]$, BDG and water applied to the ceramic surface.

	SS	df	MS	F	p
**Model (Regression)**	0.54402678	5	0.108805	9.313672	0.04783
**Total Error (Residual)**	0.03504698	3	0.011682		
**Total Adjusted**	0.57907375	8	0.072384		

Table S10. ANOVA for the mixture design using formulations composed of $[N_{1118}][C_8O_2]$, BDG and water applied to the stainless-steel surface.

	SS	df	MS	F	p
**Model (Regression)**	0.381089	5	0.076218	7.227412	0.047155
**Total Error (Residual)**	0.031637	3	0.010546		
**Total Adjusted**	0.412726	8	0.051591		

Table S11. Efficiency and pH obtained for the optimal composition of the formulation composed of $[N_{1118}][C_8O_2]$, BDG and water.

Surface	Optimal composition			Efficiency		Relative deviation (%)	pH
	BDG	$[N_{1118}][C_8O_2]$	H_2O	Experimental	Predicted		
Ceramic	13.0	7.0	80.0	0.99	1.03	3.90	8.28
Stainless-steel	13.0	8.0	79.0	1.04	1.07	2.80	8.63

Figures

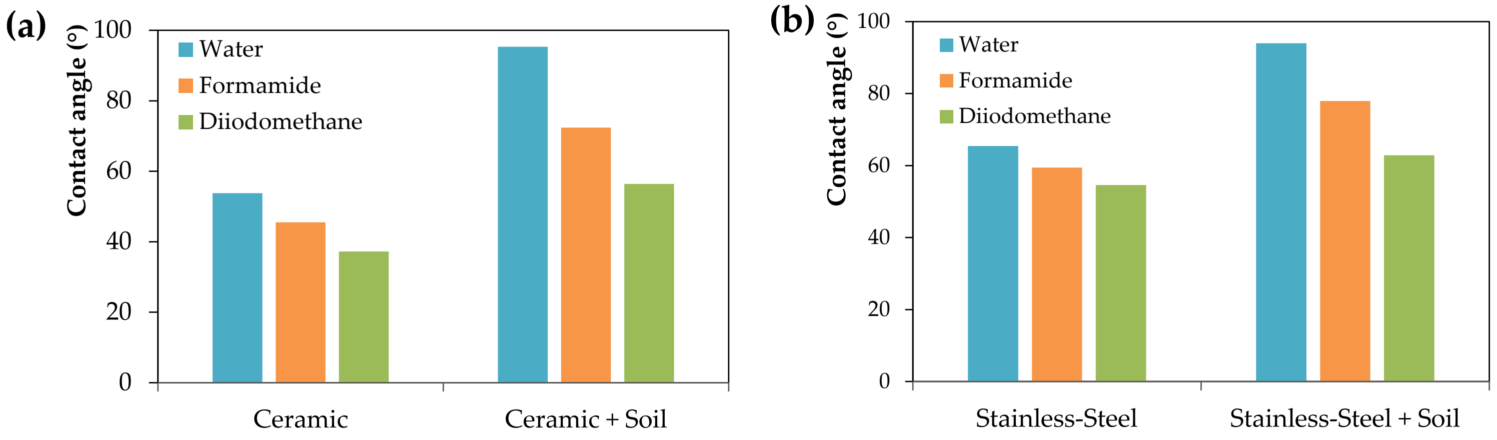

Figure S1. Contact angles of net and soiled surfaces: (a) ceramic and (b) stainless-steel.

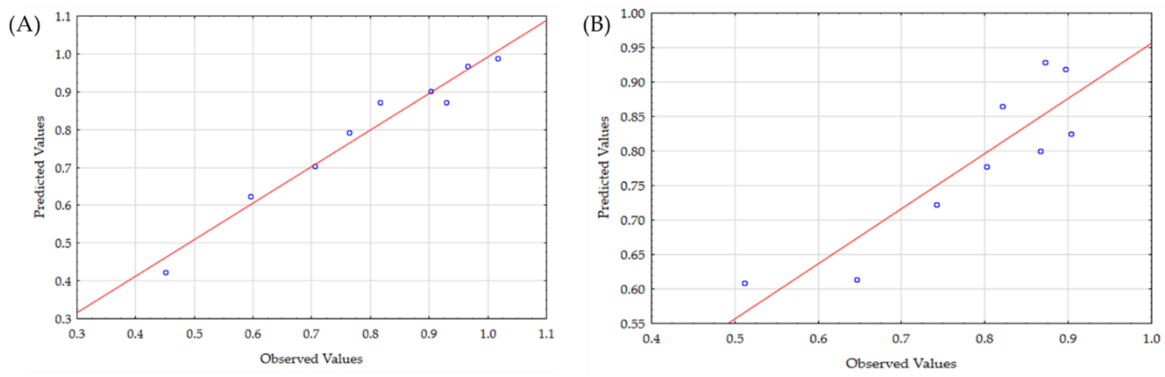

Figure S2. Predict *vs.* observed values of IPG + C_{11}-C_{13} 9EO's for soil's removal from (A) ceramic and (B) stainless-steel.

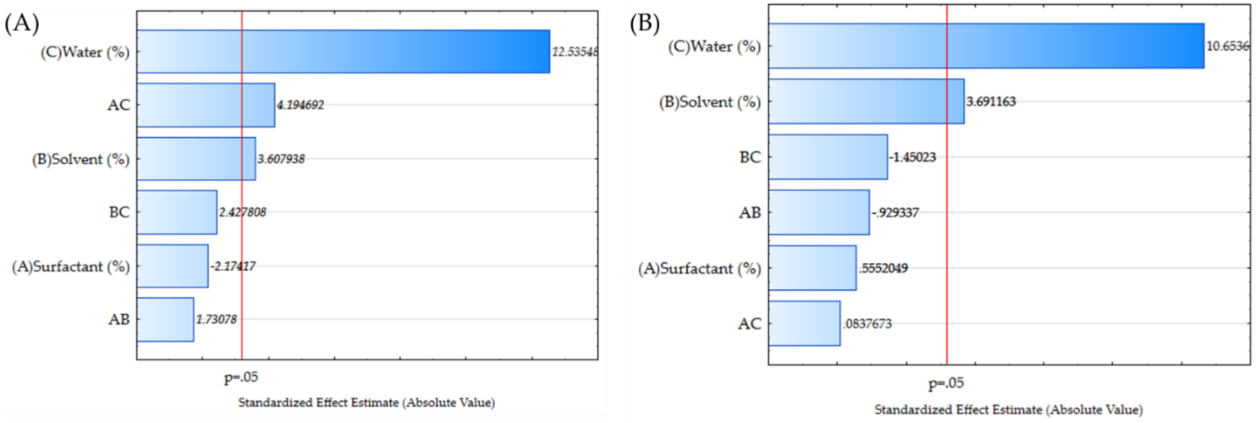

Figure S3. Pareto charts for the standardized main effects in the IPG + C$_{11}$-C$_{13}$ 9EO's mixture design for (A) ceramic and (B) stainless-steel. The vertical line indicates the statistical significance of the effects (95% of confidence).

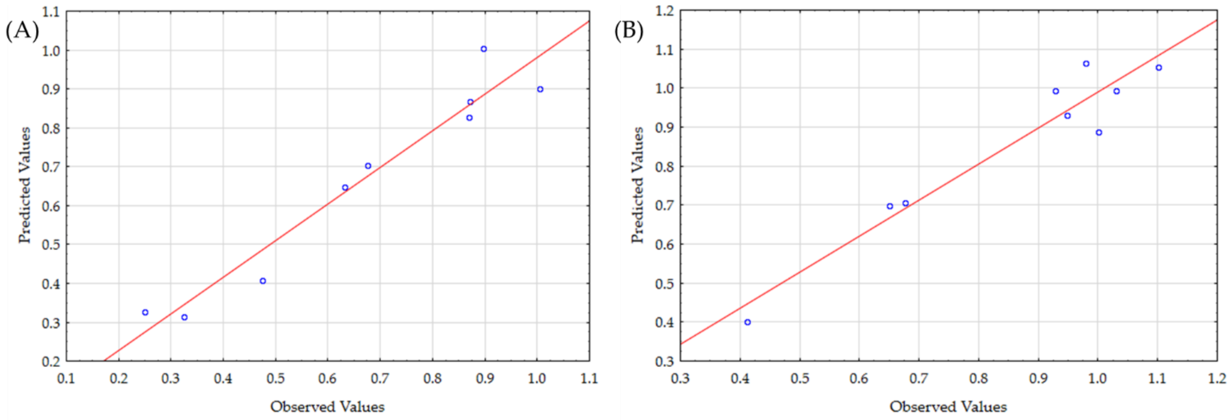

Figure S4. Predict *vs.* observed values of BDG + [N$_{1118}$][C$_8$O$_2$] for soil's removal from (A) ceramic and (B) stainless-steel.

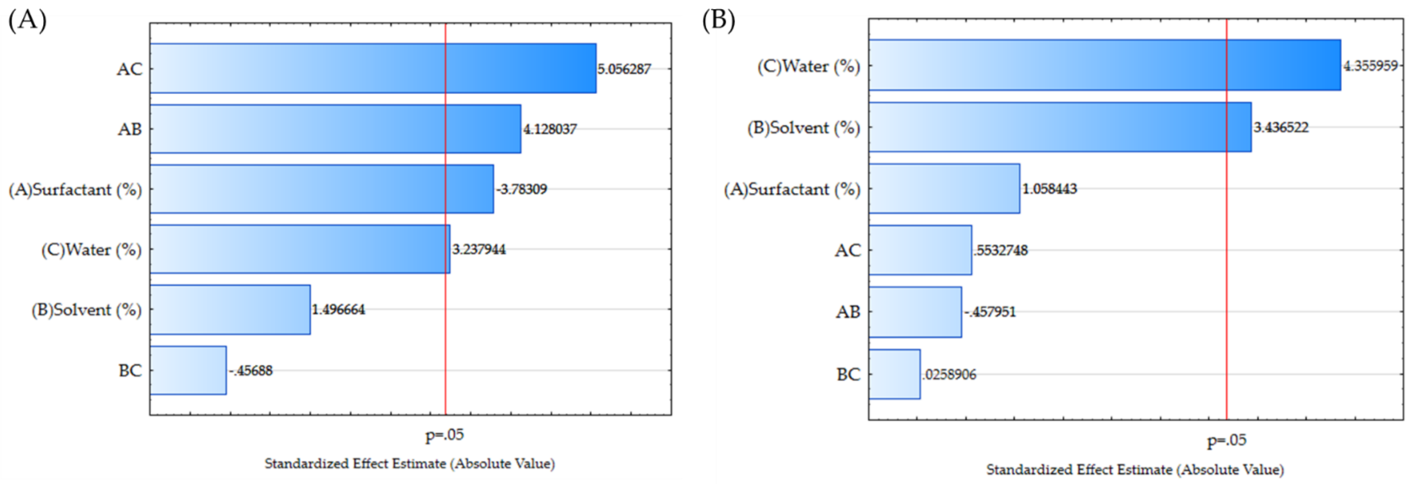

Figure S5. Pareto charts for the standardized main effects in the BDG + $[N_{1118}][C_8O_2]$ mixture design for (A) ceramic and (B) stainless-steel. The vertical line indicates the statistical significance of the effects (95% of confidence).

(A)

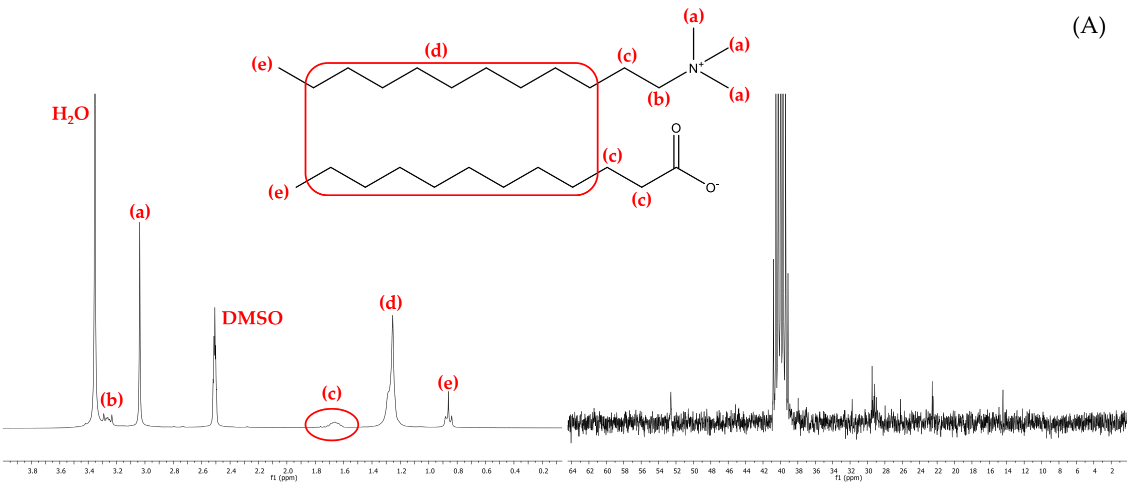
H2O
(a)
(b)
(c)
(d)
(e)
DMSO
N+
O
O-
f1 (ppm)

(B)

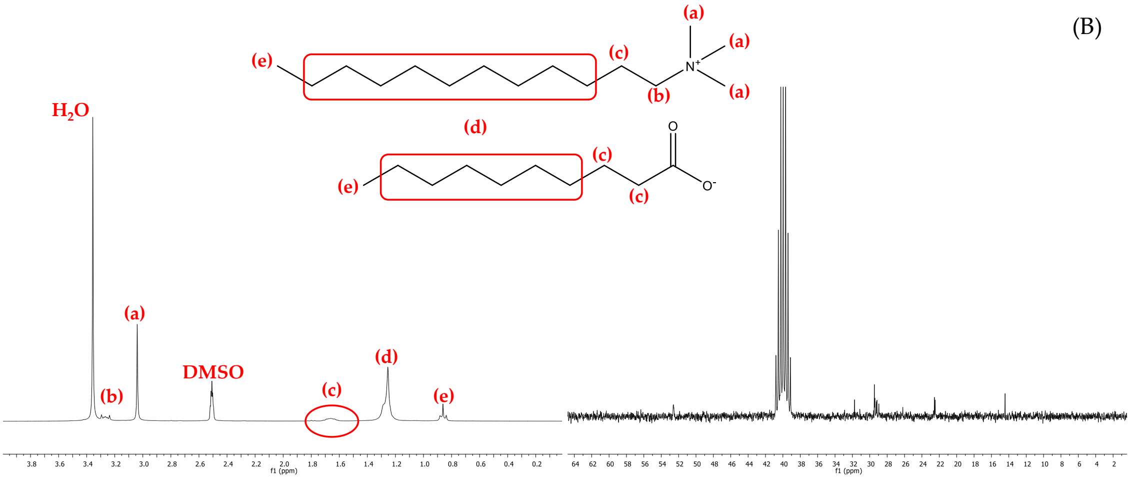
H2O
(a)
(b)
(c)
(d)
(e)
DMSO
N+
O
O-
f1 (ppm)

(C)

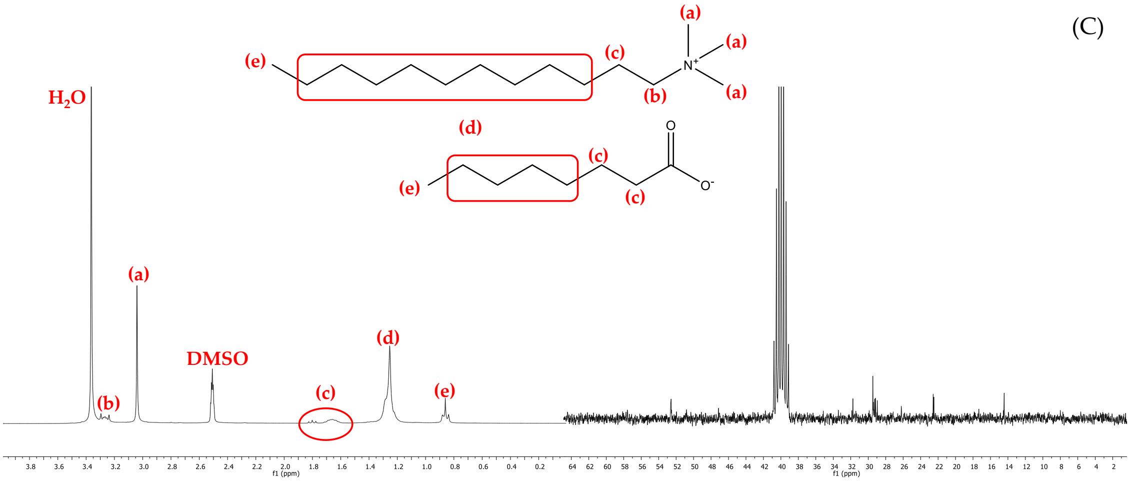
H2O
(a)
(b)
(c)
(d)
(e)
DMSO
N+
O
O-
f1 (ppm)

(D)

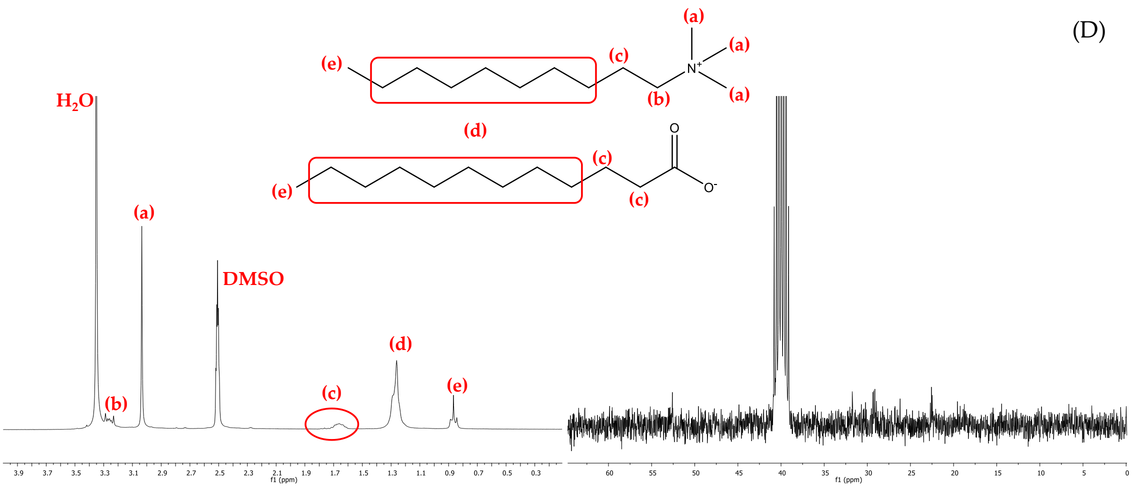
(a)
(e)
(c)
(b)
N+
(d)
O
O-
H2O
DMSO
f1 (ppm)

(E)

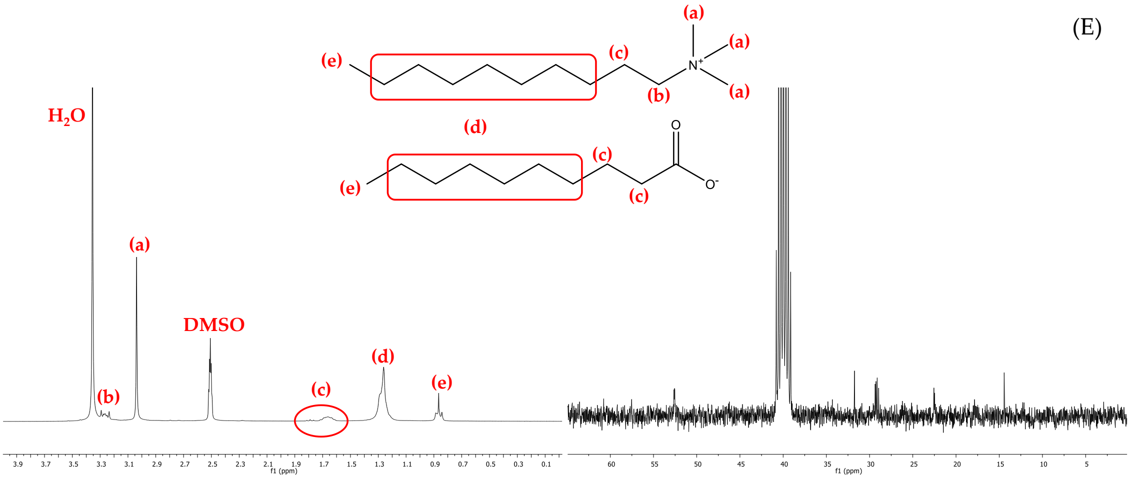
(a)
(e)
(c)
(b)
N+
(d)
O
O-
H2O
DMSO
f1 (ppm)

(F)

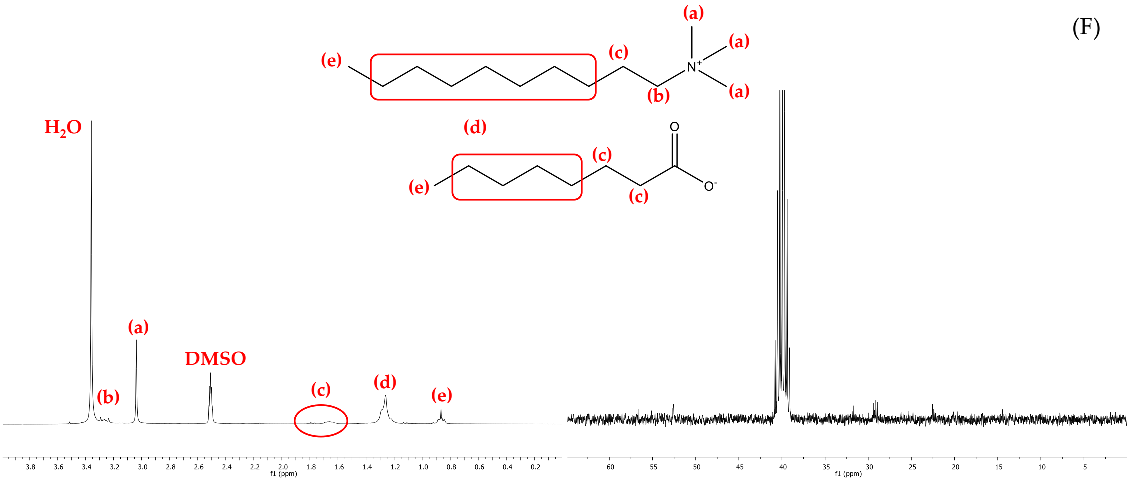
(a)
(e)
(c)
(b)
N+
(d)
O
O-
H2O
DMSO
f1 (ppm)

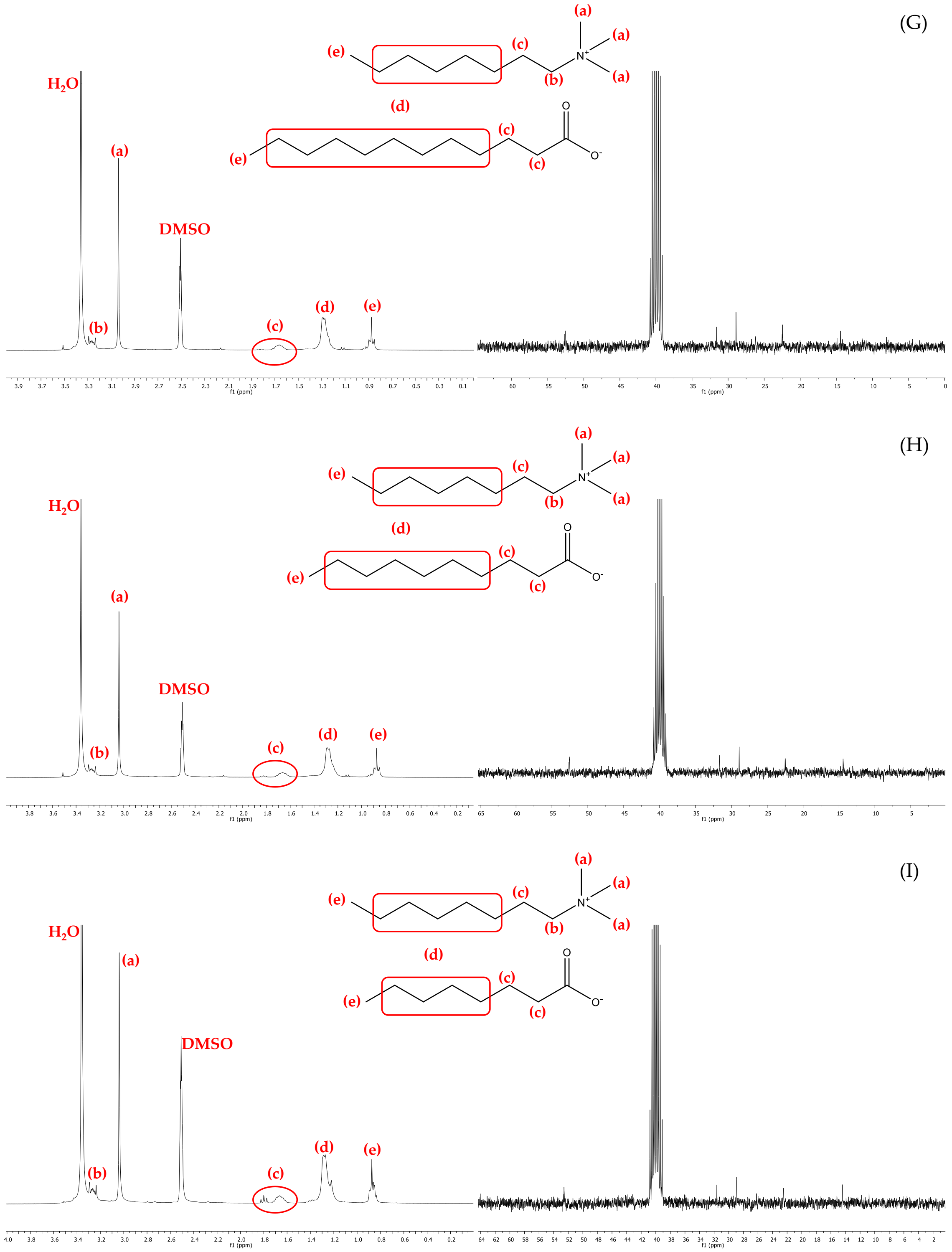

Figure S6. ^{1}H (left panel) and ^{13}C (right panel) NMR spectra in DMSO of the ionic surfactants synthesized: (A) [N$_{11112}$][C$_{12}$O$_2$]; (B) [N$_{11112}$][C$_{10}$O$_2$]; (C) [N$_{11112}$][C$_8$O$_2$]; (D) [N$_{11110}$][C$_{12}$O$_2$]; (E) [N$_{11110}$][C$_{10}$O$_2$]; (F) [N$_{11110}$][C$_8$O$_2$]; (G) [N$_{1118}$][C$_{12}$O$_2$]; (H) [N$_{1118}$][C$_{10}$O$_2$]; (I) [N$_{1118}$][C$_8$O$_2$].
